# Molecular basis for histone H3 “K4me3-K9me3/2” methylation pattern readout by Spindlin1

**DOI:** 10.1074/jbc.RA120.013649

**Published:** 2021-01-13

**Authors:** Fan Zhao, Yunan Liu, Xiaonan Su, Ji-Eun Lee, Yutong Song, Daliang Wang, Kai Ge, Juntao Gao, Michael Q. Zhang, Haitao Li

**Affiliations:** 1 MOE Key Laboratory of Protein Sciences, Beijing Advanced Innovation Center for Structural Biology, Beijing Frontier Research Center for Biological Structure, Department of Basic Medical Sciences, School of Medicine, Tsinghua University, Beijing, China; 2 MOE Key Laboratory of Bioinformatics, Bioinformatics Division, Center for Synthetic and Systems Biology, BNRist, Tsinghua University, Beijing, China; 3 Adipocyte Biology and Gene Regulation Section, Laboratory of Endocrinology and Receptor Biology, National Institute of Diabetes and Digestive and Kidney Diseases, National Institutes of Health, Bethesda, Maryland USA; 4 Department of Biological Sciences Center for Systems Biology, University of Texas at Dallas, Richardson, Texas, USA; 5 Tsinghua-Peking Center for Life Sciences, Beijing, China

**Keywords:** histone modification, Spindlin1, Tudor domain, methylation pattern, combinatorial readout, crystal structure, X-ray crystallography, epigenetics, histone methylation, gene transcription

## Abstract

Histone recognition by “reader” modules serves as a fundamental mechanism in epigenetic regulation. Previous studies have shown that Spindlin1 is a reader of histone H3K4me3 as well as “K4me3-R8me2a” and promotes transcription of rDNA or Wnt/TCF4 target genes. Here we show that Spindlin1 also acts as a potent reader of histone H3 “K4me3-K9me3/2” bivalent methylation pattern. Calorimetric titration revealed a binding affinity of 16 nm between Spindlin1 and H3 “K4me3-K9me3” peptide, which is one to three orders of magnitude stronger than most other histone readout events at peptide level. Structural studies revealed concurrent recognition of H3K4me3 and H3K9me3/2 by aromatic pockets 2 and 1 of Spindlin1, respectively. Epigenomic profiling studies showed that Spindlin1 colocalizes with both H3K4me3 and H3K9me3 peaks in a subset of genes enriched in biological processes of transcription and its regulation. Moreover, the distribution of Spindlin1 peaks is primarily associated with H3K4me3 but not H3K9me3, which suggests that Spindlin1 is a downstream effector of H3K4me3 generated in heterochromatic regions. Collectively, our work calls attention to an intriguing function of Spindlin1 as a potent H3 “K4me3-K9me3/2” bivalent mark reader, thereby balancing gene expression and silencing in H3K9me3/2-enriched regions.

Histone posttranslational modifications (PTMs), referred to as the histone codes, are essential for gene regulation and chromatin organization. They are often recognized by cognate reader modules to mediate downstream events (1). In the past two decades, more than 500 histone PTMs have been identified, among which were acetylation, crotonylation, lactylation, methylation, phosphorylation, and ubiquitination. In the meantime, a wealth of “reader” modules, such as Bromo, Chromo, Tudor, MBT, PWWP, WD40, PHD finger, DPF, YEATS, CW finger, ZZZ, and BAH domains, have been characterized to recognize histone PTMs in a type- and site-dependent manner (2, 3, 4, 5, 6).

Adding to the complexity, a rapidly increasing number of studies have demonstrated very specific roles for certain combinations of histone modifications. For instance, histone H3K4 trimethylation (H3K4me3) appears to be associated with acetylation of Lys-14, Lys-18, and Lys-23 on the same H3 tail, suggesting a concerted function of these marks in transcriptional activation (7). It has also been shown that H3S10 phosphorylation can act as a binary switch to eject HP1 from binding H3K9 trimethylated histones (H3K9me3) during M phase (8). In embryonic stem cells (ESCs), promoters of many development-associated genes are occupied by both active histone mark H3K4me3 and repressive histone mark H3K27me3 to form the so-called bivalent domains. These bivalent domains are thought to be essential for the maintenance of pluripotency by keeping genes in a “poised” state for rapid activation once signal comes (9, 10, 11).

Similarly, another bivalent histone methylation signature, H3 “K4me3-K9me3/2,” has been reported to maintain the expression of differentiation master regulatory genes at low levels in trophoblast stem cells, extraembryonic endoderm stem cells, and preadipocytes (10, 12). In addition, histone H3K9me3/2 not only exists in regions of silenced genes, but also coexists with H3K4me2 in coding regions of rDNA with active transcription (13). Consistently, the histone H3K9 demethylase PHF8 (KDM7B) has both PHD and JmjC domains and regulates rDNA transcription in nucleolus regions. The PHD domain of PHF8 recognizes H3K4me3 and promotes H3K9me2/1 demethylation by the JmjC domain *in cis* to further activate rDNA genes (14). Similarly, the H3K9me3 demethylase JMJD2A (KDM4A) contains a double Tudor domain that recognizes H3K4me3 to balance the genomic distribution of the two marks. On the one hand, the above “read-and-erase” crosstalk supports the co-existence of H3K4me3 and H3K9me3/2 under certain circumstances and, on the other hand, explains the low co-occurrence of H3K4me3 and H3K9me3/2 across our genome (15, 16). A recent study also found that H3K4me3 and H3K9me3 have some overlaps with obfuscated regulatory roles in *Neurospora crassa* (17).

Spindlin1 is a member of Spin/Ssty family and was initially identified as an abundant maternal factor in mouse oocytes and embryos at early developmental stages (18). Functional studies revealed that Spindlin1 is not only a histone reader protein that regulates downstream gene transcription (19, 20), but also is involved in chromosome segregation during mitotic or meiotic cell division (21, 22, 23). In addition, Spindlin1 is overexpressed in a number of cancers (19, 24), suggesting its tumor promoter function. Previous studies reported that Spindlin1 recognizes histone H3K4me3 and H3 “K4me3-R8me2a” (R8me2a, asymmetric dimethylation of arginine 8) methylation patterns, which promotes gene expression of rDNA, Wnt/TCF4 target genes, and MAZ (Myc-associated zinc finger protein) regulated genes (20, 25, 26). Structural studies have revealed that Spindlin1 consists of three Spin/Ssty motifs that adopt a Tudor-like β-barrel fold (27), of which the second Tudor recognizes H3K4me3 or H4K20me3 and the first Tudor module recognizes H3R8me2a, rendering Spindlin1 a multifunctional histone reader for H3K4me3, H4K20me3, and H3“K4me3-R8me2a”(25, 26, 28).

Given the function of the bivalent histone H3 “K4me3-K9me3/2” methylation pattern in regulating genes such as differentiation master genes in early development and rDNA, it raises an intriguing question regarding the occurrence of its downstream effectors (20, 26, 29). Here we characterized Spindlin1 as a potent reader for bivalent H3 “K4me3-K9me3/2” methylation pattern. Our structural studies revealed combinatorial readout of K4me3 and K9me3/2 by Spindlin1. Moreover, our ChIP-Seq and ChIP-qPCR assays revealed genomic colocalization of Spindlin1, H3K4me3, and H3K9me3. In some regions, Spindlin1 and H3K4me3 coexist as sharp peaks in the widely distributed H3K9me3 background, suggesting that Spindlin1 is recruited to chromatin primarily through H3K4me3. The fact that additional H3K9me3/2 further boosts a stable association of Spindlin1 with H3 tail calls attention to a role of Spindlin1 in dynamic regulation of gene expression in H3K9me3/2-enriched regions.

## Results

### Spindlin1 is a potent histone reader for bivalent H3 “K4me3-K9me” methylation pattern

**Figure 1:**
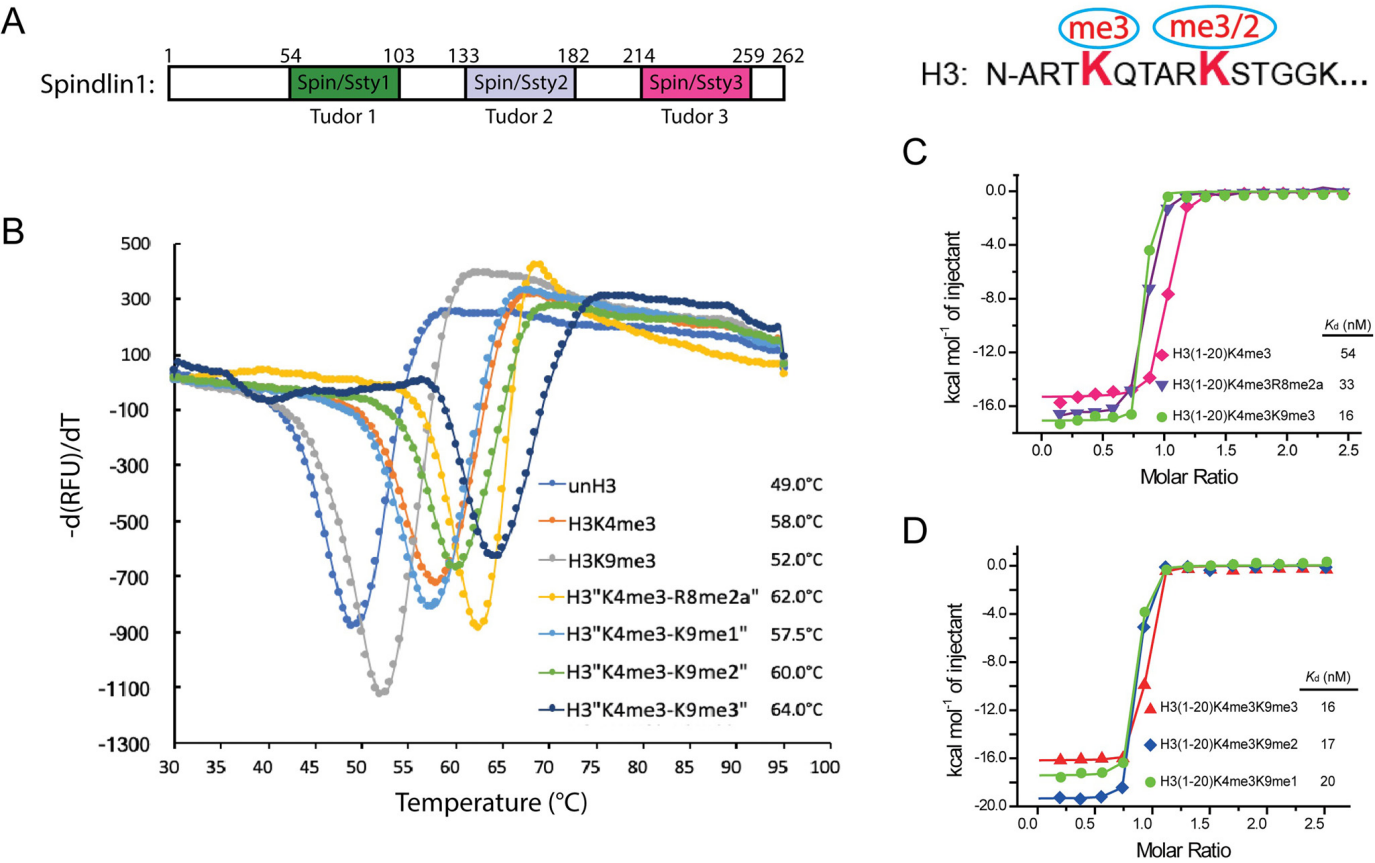
**Affinity characterization of Spindlin1 as a bivalent histone H3 “K4me3-K9me3/2” methylation pattern reader.***A*, *left*, domain architecture of Spindlin1. The three Spin/Ssty repeats are colored *green*, *gray*, and *pink*, respectively. *Right*, sequence and methylation pattern of H3 “K4me3-K9me3/2”. *B*, TSA melting curves of Spindlin1_50–262_ with histone H3 peptides in different methylation states. *C*, ITC fitting curves of Spindlin1_50–262_ with histone H3K4me3, H3 “K4me3-R8me2a,” and H3 “K4me3-K9me3” peptides. *D*, ITC fitting curves of Spindlin1_50–262_ with histone H3K4me3 peptides of different K9 methylation states.

Spindlin1 has been characterized as a histone reader for a *cis*-tail H3 “K4me3-R8me2a”methylation pattern in processes of Wnt signaling and rDNA transcription (25, 29). Here we wanted to ask if the histone H3 “K4me3-K9me3/2” methylation pattern could be recognized by Spindlin1 or not (Fig. 1*A*).

First, we conducted thermal shift assays (TSA) using Spindlin1_50–262_ mixed with different histone H3 methylation peptides. Surprisingly, H3_1–20_ “K4me3-K9me3” peptide displayed the most pronounced stabilization effect with a melting temperature (*Tm*) of 64.0°C, which is 2.0°C, 4.0°C, 6.5°C, 6.0°C, 12.0°C, and 15.0°C greater than those of H3_1–20_ “K4me3-R8me2a,” H3_1–20_ “K4me3-K9me2,” H3_1–20_ “K4me3-K9me1,” H3_1–20_ K4me3, H3_1–20_ K9me3, and unmodified H3_1–20_, respectively (Fig. 1*B*). These results suggest that H3K9me3/2 is a recognition mark for Spindlin1 and its combination with H3K4me3 can significantly stabilize H3-Spindlin1 association.

Next we used isothermal titration calorimetry (ITC) to quantitatively characterize these interactions. Compared with the binding affinity of Spindlin1_50–262_ for H3_1–20_ K4me3 peptide (*K_d_* = 54 nm), the binding affinity is enhanced to 33 nm for H3 “K4me3-R8me2a” and 16 nm for H3 “K4me3-K9me3” methylation pattern (Fig. 1*C*). Moreover, we measured binding affinities of 16 nm, 17 nm, and 20 nm between Spindlin1_50–262_ and H3 “K4me3-K9me3,” “K4me3-K9me2,” and “K4me3-K9me1” peptides, respectively (Fig. 1*D*). The nanomolar binding affinity between Spindlin1_50–262_ and H3_1–20_ “K4me3-K9me3/2” peptides, along with the pronounced thermal stabilization effect of the complex, suggests that Spindlin1 is a potent reader for histone H3 “K4me3-K9me3/2” bivalent methylation patterns.

### Overall structure of Spindlin1 bound to H3“K4me3-K9me3/2” peptides

**Figure 2:**
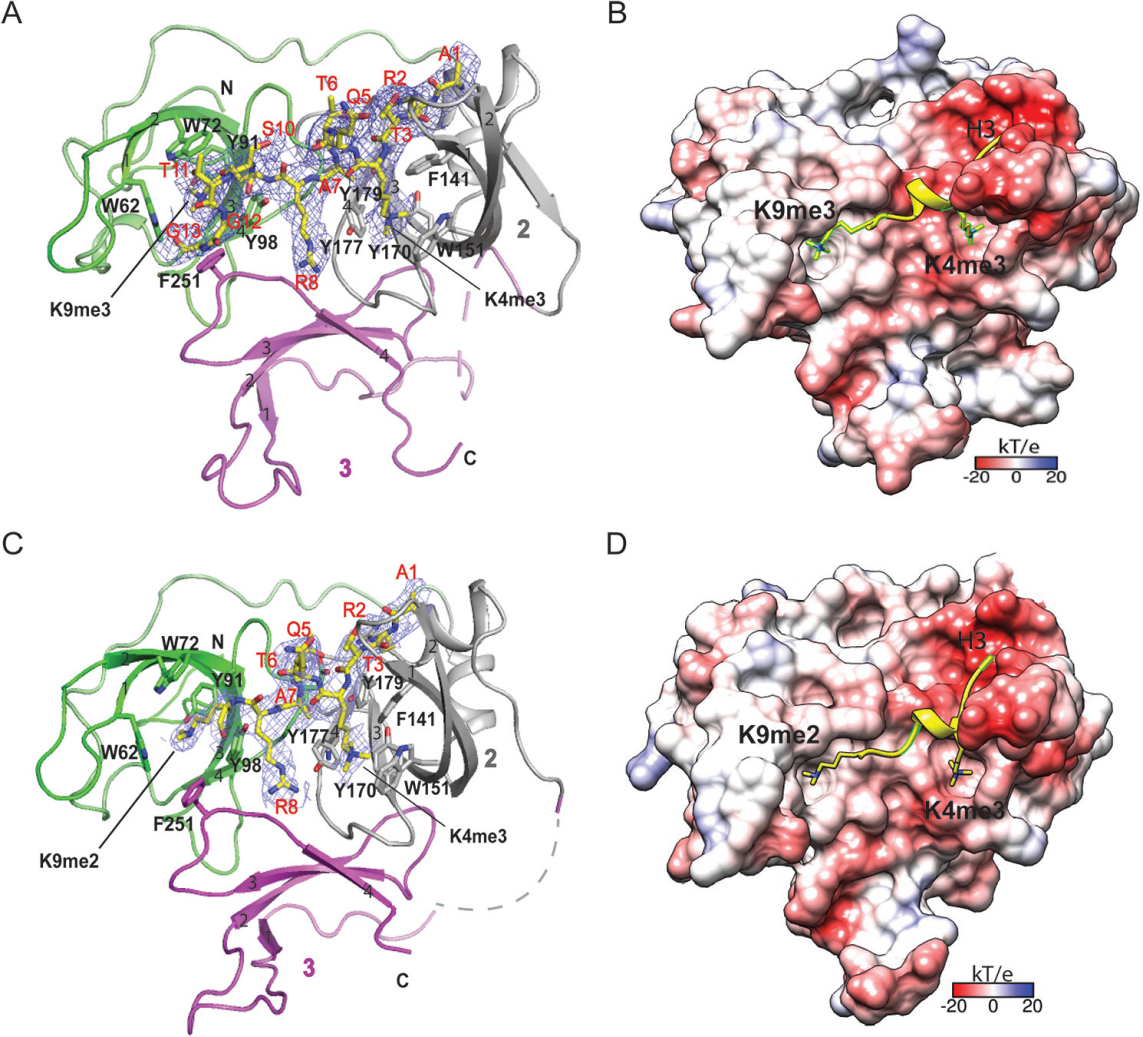
**Overall structures of Spindlin1_45–262_ with bivalent histone H3 “K4me3-K9me3/2” peptides.***A*, ribbon view of Spindlin1_45–262_ bound to H3 “K4me3-K9me3” peptide highlighted as *yellow sticks*. Residues Ala-1 to Gly-13 of H3 “K4me3-K9me3” peptide are well traced as shown by the 2*F_o_* − *F_c_* omit map countered at 1.5 σ level. *B*, electrostatic surface views of Spindlin1_45–262_ bound to H3 “K4me3-K9me3” peptide shown as *yellow ribbon* with highlighted K4me3 and K9me3 in *sticks*. Electrostatic potential is expressed as a spectrum ranging from −20 kT/e (*red*) to +20 kT/e (*blue*). *C*, ribbon view of Spindlin1_45–262_ bound to H3 “K4me3-K9me2” peptide highlighted as *yellow sticks*. Residues Ala-1 to Lys-9 of H3 “K4me3-K9me2” peptide are well traced as shown by the 2*F_o_* − *F_c_* omit map countered at 1.5 σ level. *D*, electrostatic surface views of Spindlin1_45–262_ bound to H3 “K4me3-K9me2” peptide shown as *yellow ribbon* with highlighted K4me3 and K9me2 in *sticks*. Electrostatic potential is expressed as a spectrum ranging from −20 kT/e (*red*) to +20 kT/e (*blue*).

To explore the underlying molecular basis for H3 “K4me3-K9me3/2” recognition by Spindlin1, we first solved the crystal structure of Spindlin1 bound to H3_1–15_ “K4me3-K9me3” peptide at 3.1 Å (Table 1). Similar to previous studies, Spindlin1 is composed of three Tudor-like Spin/Ssty repeats that are organized in a triangular architecture (Fig. 2*A*). We were able to trace H3 residues Ala-1 to Gly-13 according to the electron density map (Fig. 2*A*). The H3 peptide lies in the negative charge surface of Spindlin1 with an induced short α-helix encompassing Lys-4 to Ala-7 (Fig. 2*B*). In the complex structure, the two methylation marks, H3K4me3 and H3K9me3, are inserted into the aromatic pockets of the second and the first Tudor domains of Spindlin1, respectively. The aromatic cage in Tudor 2 is formed by Phe-141, Trp-151, Tyr-170, and Tyr-177, and the aromatic cage in Tudor 1 is composed of Trp-62, T rp-72, Tyr-91, Tyr-98, and Phe-251 (Fig. 2, *A* and *B*).

**Table 1:** Data collection and refinement statistics

	Spindlin1-H3 “K4me3-K9me3”	Spindlin1-H3 “K4me3-K9me2”
**PDB code**	7BQZ	7BU9
**Data collection**		
Wavelength (Å)	0.9792	0.9792
Space group	P2_1_	P2_1_
Cell dimensions		
*a*, *b*, *c* (Å)	40.5, 142.7, 132.3	40.4, 143.5, 129.6
α, β, γ (°)	90, 95.4, 90	90, 95.8, 90
Resolution (Å)	50–3.10 (3.15–3.10)^a^	50–3.50 (3.56–3.50)
*R*_merge_ (%)	13.0 (64.7)	15.3 (49.0)
*I*/σ*I*	10.8 (1.7)	8.0 (1.9)
Completeness (%)	98.5 (99.2)	98.9 (99.9)
Redundancy	3.5 (3.6)	3.5 (3.6)
**Refinement** (F > 0)		
Resolution (Å)	48.4–3.1	48.0–3.5
No. of reflections	26,589	18,458
*R*_work_/*R*_free_ (%)	22.4/26.4	24.6/26.4
No. of atoms		
Protein	6709	6559
Ligand	355	330
*B*-factors (Å^2^)		
Protein	69.4	76.7
Ligand	72.3	77.6
R.m.s. deviations		
Bond lengths (Å)	0.003	0.004
Bond angles (°)	0.885	0.770


a Values in parentheses are for highest-resolution shell.

Besides, we also solved the crystal structure of Spindlin1 bound to H3_1–15_ “K4me3-K9me2” peptide at 3.5 Å (Table 1). H3 residues Ala-1 to Lys-9 were modeled in the complex structure (Fig. 2*C*). Consistently, the H3K4me3 and H3K9me2 marks are positioned into the aromatic cages of the second and the first Tudor domains of Spindlin1 (Fig. 2*D*). We measured a root mean square deviation (r.m.s.d.) of 0.458 Å between the two superimposed complexes.

### Details of Spindlin1-H3 “K4me3-K9me3” interaction

**Figure 3:**
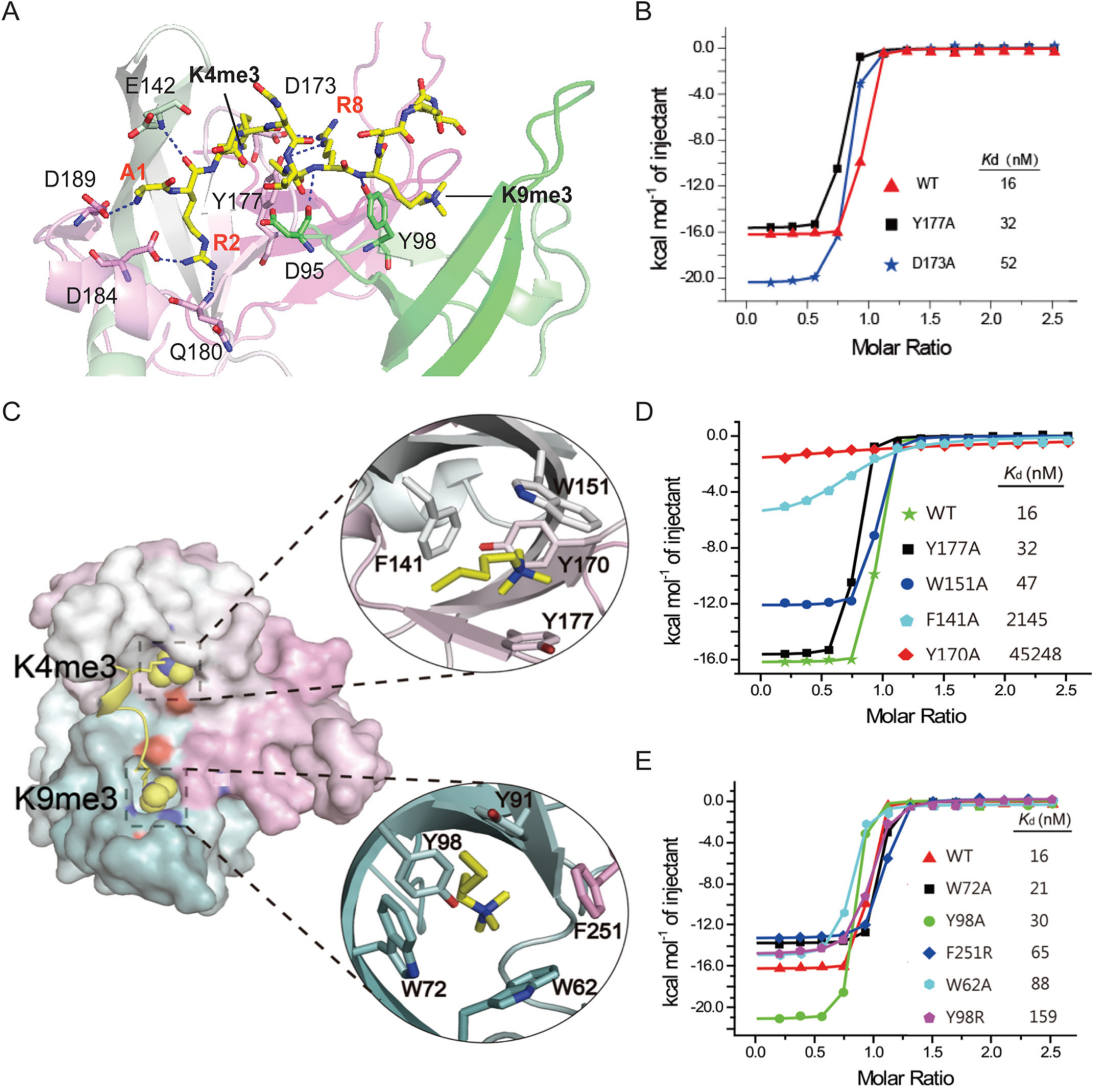
**Details of H3 “K4me3-K9me3” methylation pattern recognition by Spindlin1 and mutagenesis studies.***A*, stereo view of polar contacts between Spindlin1 and H3 “K4me3-K9me3” peptide. The H3 peptide and key Spindlin1 residues involved in histone recognition are shown as sticks. *Blue dashes* represent hydrogen bonds or salt bridges. *B*, ITC fitting curves of mutant Spindlin1 titrated with H3 “K4me3-K9me3” peptide. *C*, encapsulation of K4me3 and K9me3 by Spindlin1 pockets 2 and 1. Spindlin1 is shown in surface mode with Tudors 1, 2, and 3 colored *green*, *gray*, and *pink*, respectively. Close-up views of K4me3 (*top*) and K9me3 (*bottom*) pockets. *D* and *E*, ITC fitting curves of Spindlin1 K4me3 (*D*) and K9me3 (*E*) pocket mutants titrated with H3 “K4me3-K9me3” peptide.

In the cocrystal structure, the H3 segment is anchored on the surface of Spindlin1 by a network of polar interactions, including eight direct hydrogen bonds or salt bridges between Spindlin1 and the H3 peptide. These interactions are contributed by residue pairs H3A1:D189, H3R2:D184, H3R2:Q180, H3R2:E142, H3R8:D95, H3R8:Y177, H3R8:D173 and H3K9:Y98 (Fig. 3*A*). In particular, H3R8 is stabilized by Asp-173 and Tyr-177, whose binding contributions are validated by an affinity drop of 2-fold for Y177A and 3-fold for D173A (Fig. 3*B*). The two methylation marks H3K4me3 and H3K9me3 are encapsulated by their respective aromatic pockets with buried surface areas of 214 Å^2^ and 236 Å^2^. In the Spindlin1-H3 “K4me3-K9me2” complex, the buried surface area is 231 Å^2^ for the H3K9me2 mark. Both aromatic pockets recognize methylated lysine 4 or 9 through cation-π and methyl-π interactions (Fig. 3*C*).

Next, we generated point mutants of the aromatic cage residues in Tudors 2 and 1. As expected, single alanine mutation of the aromatic residues in Tudor 2 resulted in an affinity reduction of ∼2800-fold and ∼130-fold for Y170A and F141A, respectively (Fig. 3*D*). By contrast, point mutation of most aromatic residues in pocket 1 displayed mild binding loss (∼1- to 5-fold), excepted that Y98R displayed 10-fold binding reduction (Fig. 3*E*). Collectively, these data suggest that recognition of K4me3 by pocket 2 dominates binding, whereas K9me3 recognition by pocket 1 enhances binding and enables a more thermal stable complex formation.

### Comparison of H3 K9me3/2 and R8me2a readout by Spindlin1

**Figure 4:**
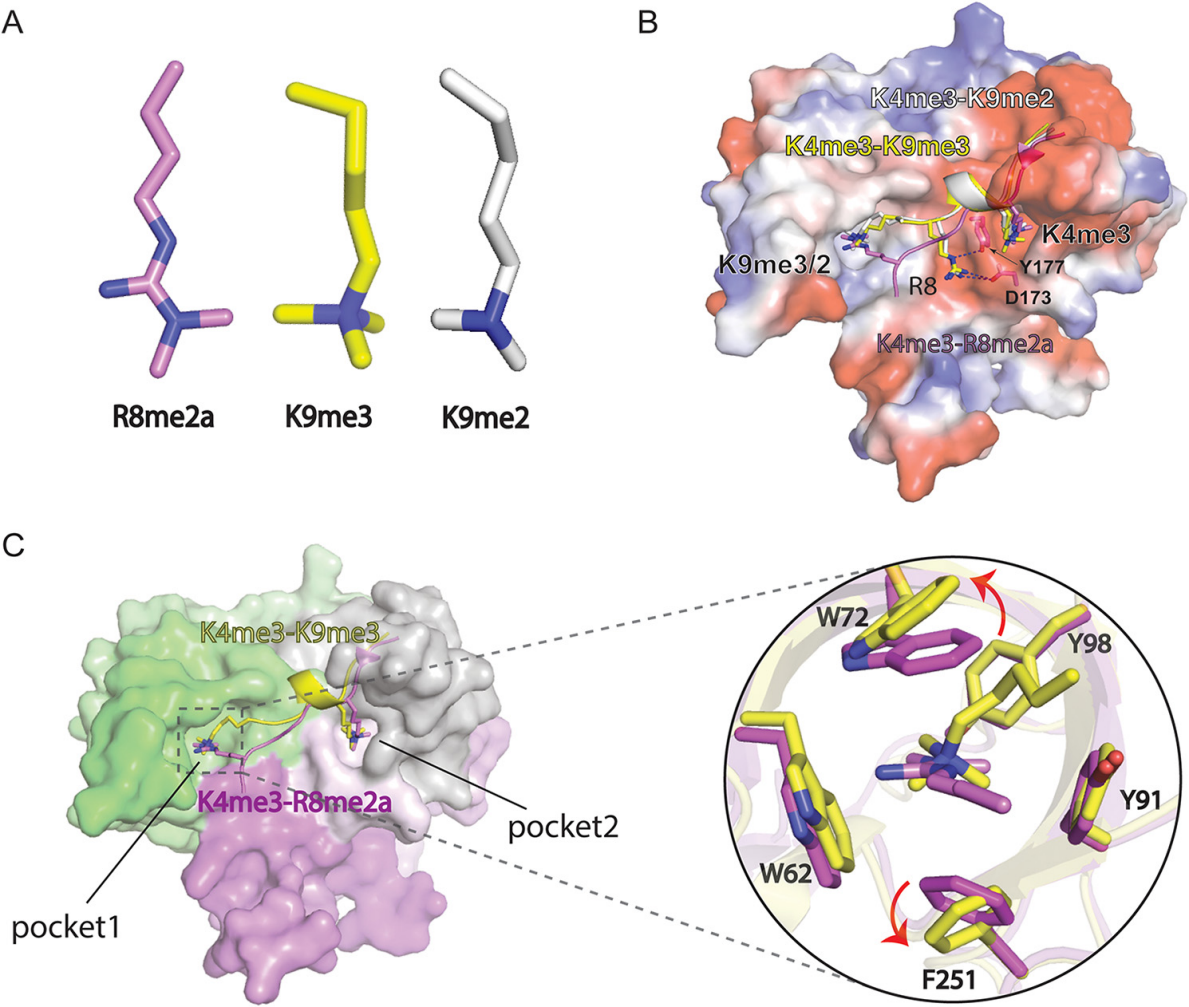
**Structural comparison of Spindlin1 bound to H3 “K4me3-K9me3/2” with Spindlin1 bound to H3 “K4me3-R8me2a.”** A, stereochemical structures of R8me2a (*magenta*), K9me3 (*yellow*), and K9me2 (*white*). *B*, structural alignment of Spindlin1 bound to H3 “K4me3-K9me3” (*yellow*), H3 “K4me3-K9me2” (*white*) and H3 “K4me3-R8me2a” (*magenta*). Spindlin1 was shown as electrostatic surface view. H3 peptides are shown as ribbon views with K4me3, K9me3, K9me2, and R8 are shown as sticks. *C*, *left*, surface view of Spindlin1 with Tudors 1, 2, 3 colored in *green*, *gray*, and *magenta*. Ribbon views of H3 “K4me3-R8me2a” (*magenta*) and H3 “K4me3-K9me3” (*yellow*) peptides with highlighted K4me3, K9me3, and R8me2a shown as sticks. (*right*) Structural alignment and close-up views of aromatic pocket 1 of Spindlin1 with K9me3 and R8me2a inserted.

Aside from the H3 peptide, the overall structure of the H3 “K4me3-K9me3” complex is essentially the same as that of the H3 “K4me3-R8me2a” complex with a superimposition root mean square deviation of 0.606 Å. It is interesting that the same pocket of Spindlin1 is responsible for both H3R8me2a and H3K9me3/2 mark readout (Fig. 4*A*). In the H3 “K4me3-R8me2a” complex structure reported previously, the backbone of H3 peptide is rather extended, and the K4me3 and R8me2a marks are perpendicularly inserted into their respective reader pocket. By contrast, to ensure proper registration of H3K9me3/2 into pocket 1, the H3 peptide takes on a more crouched conformation with an induced α-helix in the middle and the H3K9me3/2 mark is inserted into aromatic cage from the side (Fig. 4*B*). Correspondingly, unmodified H3R8 side chain flips away from pocket 1 and is stabilized by hydrogen bonding interactions with Asp-173 and Tyr-177 (Fig. 4*B*). The aromatic cage of Tudor 1 is reorganized to fit H3K9me3 insertion, which involves in flipping of Trp-72 and Phe-251 side chains (Fig. 4*C*). Compared with H3R8 insertion from the top, the side insertion of H3K9me3 allows a better encapsulation by the aromatic pocket, which is consistent with a larger buried surface area of 236 Å^2^ for K9me3 as compared with an area of 216 Å^2^ for R8me2a (Fig. 4*C*). Furthermore, the intimate encapsulation of K9me3/2 mark by aromatic pocket 1 of Spindlin1 is also consistent with a stable and high-affinity association of Spindlin1 with the H3 “K4me3-K9me3/2”peptide.

### Spindlin1 colocalizes with bivalent H3 “K4me3-K9me3”chromatin regions

**Figure 5:**
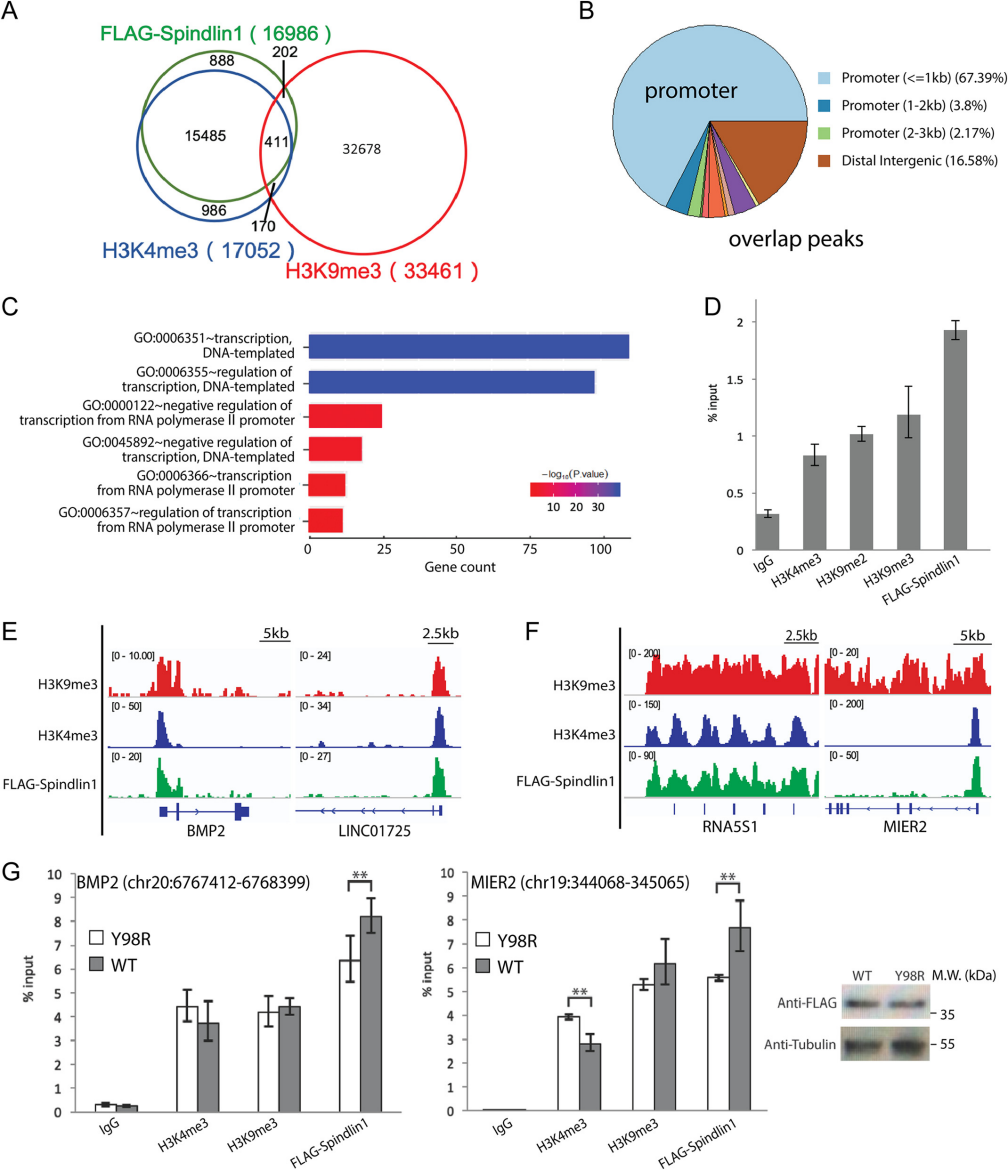
**Colocalization studies of Spindlin1 and H3 “K4me3-K9me3” methylation pattern.***A*, Venn diagram of the overlap of annotated Spindlin1, H3K4me3, and H3K9me3 peaks. *B*, genomic distribution of overlap peaks. *C*, GO analysis of biological process in overlapping peaks. GO enrichment was analyzed with Cluster Profiler. *D*, ChIP-qPCR analysis using FLAG beads, H3K4me3, H3K9me2, H3K9me3 antibodies, and IgG control to probe the enrichment of FLAG-Spindlin1 and H3 “K4me3-K9me3/2” methylation pattern in the promoter regions of rDNA genes in HEK293 cells. *E* and *F*, example browser views of the co-occurrence of Spindlin1, H3K4me3, and H3K9me3 peaks. *G*, ChIP-qPCR analysis using FLAG beads, H3K4me3, H3K9me3 antibodies, and IgG control to probe the enrichment of FLAG-Spindlin1 (WT or Y98R), H3K4me3, and H3K9me3 in two genomic overlap regions. *Error bars*, S.D. **, *P* < 0.05, two-tailed Student's *t* test. *Right*, a Western blotting with anti-FLAG and anti-tubulin antibodies shows that Spindlin1 WT and Y98R are expressed at similar level.

To investigate the genomic distribution of Spindlin1, H3K4me3, and H3K9me3, we performed ChIP followed by sequencing (ChIP-Seq) in HEK293 cells with overexpressed FLAG-Spindlin1. Peak calling and analysis of the overlap peaks showed a strong correlation of Spindlin1 and H3K4me3, in which 93.6% Spindlin1 peaks overlap with H3K4me3, consistent with previous findings (24). Although on the whole H3K4me3 and H3K9me3 are distributed in distinct genomic regions with opposing roles in gene regulation, we found 3.4% of all H3K4me3 peaks overlap with H3K9me3. Among these overlapping peaks, about 411 peaks (∼70.7%) are occupied by Spindlin1 (Fig. 5*A*). Genomic distribution analysis further revealed that >70% of the overlap peaks are located in the gene promoter regions (Fig. 5*B*).

We characterized 322 genes in the 411 overlap peaks. Intriguingly, GO analysis on biological process showed that more than 40% of the genes are clustered in gene transcription and its regulation, and they code for zinc finger proteins, epigenetic enzymes and effectors, and so on (Fig. 5*C*). Apart from the genes clustered in the gene regulation, about 5.6% of the overlap genes are noncoding RNAs, including microRNAs and long noncoding RNAs. Furthermore, our ChIP-qPCR studies showed that H3 “K4me3-K9me3/2” and Spindlin1 also co-enriched in promoter regions of rDNA gene, consistent with a previously reported function of Spindlin1 in nucleolus (Fig. 5*D*).

We next analyzed the width of H3K9me3 peaks in overlap regions, ranging from 300 to more than 75,000 base pairs (bp). Taking H3K9me3 peak width of 3000 bp as a criterion, we could divide the overlap peaks into two groups: H3K9me3 sharp (<3000 bp) and H3K9me3 broad (>3000 bp) regions. The H3K9me3 sharp group constitutes 67.9% of all overlap peaks marked by H3K9me3, H3K4me3, and Spindlin1. Example genes include noncoding RNA gene LINC01725 and developmental signal protein gene BMP2 (Fig. 5*E*). The rest of the overlap peaks are H3K9me3 broad group, among which include the rDNA gene RNA5S1 and transcription repressor gene MIER2 (Fig. 5*F*).

To validate the role of H3K9me3 recognition in this binding event, we performed ChIP-qPCR for WT Spindlin1 and the H3K9me3 pocket mutant Y98R at two genome sites, the BMP2 promoter region with sharp H3K9me3 peak, and the MIER2 promoter region with broad H3K9me3 peak. As shown in Fig. 5*G*, when FLAG-Spindlin1 WT and Y98R are expressed at a similar level in HEK293 cells, we found that the enrichment of mutant Y98R in these two regions was mildly decreased compared with that of WT, suggesting a role of K9me3 recognition for robust recruitment of Spindlin1 *in vivo*.

Considering that Spindlin1 has a high correlation with H3K4me3 in genomic distribution and nanomolar-level binding affinity for histone H3 “K4me3-K9me3/2” methylation pattern, we believe that Spindlin1 is likely a downstream effector of H3K4me3 created in H3K9me3/2-enriched regions, and the H3 “K4me3-K9me3/2” pattern enables a stable engagement of Spindlin1 with chromatin to regulate target gene expression.

### Spindlin1 is a unique histone reader for the H3 “K4me3-K9me3/2” methylation pattern

**Figure 6:**
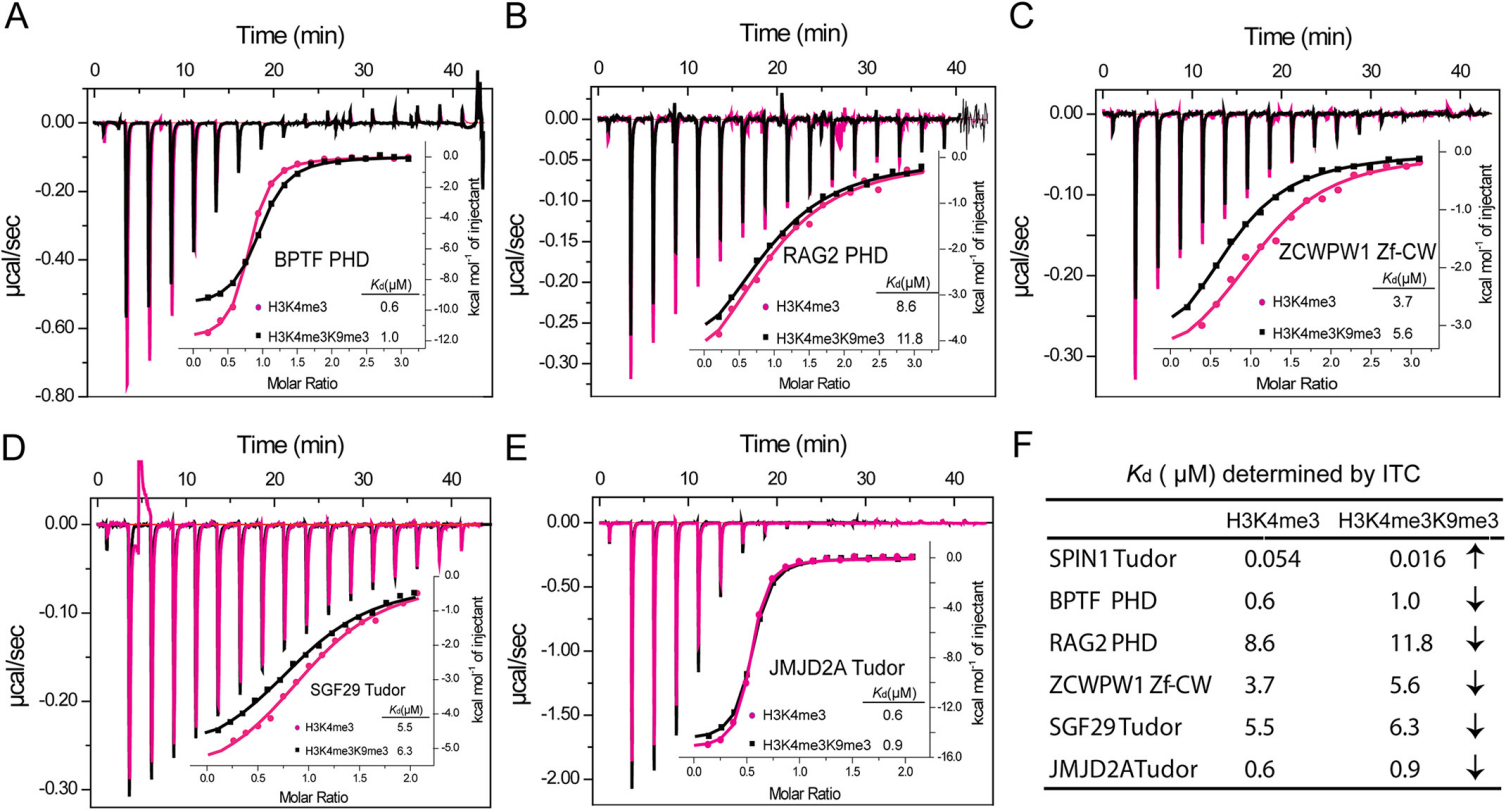
**Effects of H3K9me3 on the affinity of several canonical readers for H3K4me3.***A* and *B*, ITC fitting curves of PHD domains of BPTF (*A*) and RAG2 (*B*) titrated with H3K4me3 and H3 “K4me3-K9me3” peptides. *C*, ITC fitting curves of ZCWPW1 Zf-CW domain titrated with H3K4me3 and H3 “K4me3-K9me3” peptides. *D* and *E*, ITC fitting curves of Tudor domains of SGF29 (*D*) and JMJD2A (*E*) titrated with H3K4me3 and H3 “K4me3-K9me3” peptides. *F*, summary of *K_d_* values determined by ITC. The *upward arrow* indicates an increase in binding affinity, and the *downward arrow* indicates a decrease in binding affinity.

Previous studies have revealed that H3K4me3 could be recognized by a series of modules, such as Chromo, PHD, Tudor, and Zf-CW domains. Here we selected representative H3K4me3 readers, namely the PHD domains of BPTF and RAG2, the Zf-CW domain of ZCWPW1, and the double Tudor domains of SGF29 and JMJD2A and compared their binding affinities toward H3K4me3 and H3 “K4me3-K9me3.” Using ITC, we measured micromolar-level binding affinities of these readers for H3K4me3 and H3 “K4me3-K9me3” peptides with 0.6 and 1.0 μm for BPTF (Fig. 6*A*), 8.6 and 11.8 μm for RAG2 (Fig. 6*B*), 3.7 and 5.6 μm for ZCWPW1 (Fig. 6*C*), 5.5 and 6.3 μm for SGF29 (Fig. 6*D*), and 0.6 and 0.9 μm for JMJD2A (Fig. 6*E*), respectively. We found that the existence of H3K9me3 is slightly tolerated by the nonspindlin1 H3K4me3 readers, with about 1.1- to 1.7-fold reduced affinity for the H3 “K4me3-K9me3” pattern (Fig. 6*F*). In contrast, H3K9me3 further promotes H3-Spindlin1 interaction by both 3-fold *K_d_* increase to 16 nm and by 6°C thermostability shift to 64°C, demonstrating that Spindlin1 is a unique and potent histone reader for bivalent histone H3 “K4me3-K9me3” pattern.

## Discussion

Histone PTMs and their readout play critical roles in regulating chromatin-templated cellular processes, such as transcription, replication, and mitosis. In fact, histone PTMs often exist in a combinatorial pattern, which calls for a mechanism of combinatorial readout by paired reader modules (30). Here we found that Spindlin1 can serve as a potent reader for histone H3 “K4me3-K9me3/2” methylation pattern. Our structural studies revealed that H3K4me3 and H3K9me3/2 are concurrently recognized by the aromatic cages of Tudors 2 and 1 of Spindlin1, respectively. Binding and mutagenesis studies suggest that H3K4me3 serves as the binding “hot spot” for efficient H3-Spindlin1 recognition (Fig. 3, *D* and *E*), and additional H3K9me3/2 enables a robust association with enhanced affinity and thermal stability (Fig. 1, *B* and *C*) (31).

The genome-wide and dynamic distributions of many histone PTMs have been well documented. Interestingly, histone H3K9me3–dependent heterochromatin constitutes a major barrier of cell fate transition, and it undergoes dramatic reprogramming during early development (32). Although in most cases, the genomic distributions of H3K4me3 and H3K9me3 are quite distinct, here we revealed that a small portion of the ChIP-Seq peaks displayed coexistence of H3K4me3 and H3K9me3 as well as Spindlin1 (Fig. 5*A*). These regions include genes of rDNA, zinc finger protein, long noncoding RNAs and developmental signal proteins. Remarkably, these genes are clustered in biological processes such as transcription and its regulation (Fig. 5*C*), suggesting the functional importance of the H3 “K4me3-K9me3” bivalent mark and its readout.

It has been reported that most histone H3K9 methyltransferases, including SETDB1, G9A, GLP, and SUV39H1, were sensitive to preexisting H3K4me3 mark and their enzymatic activities were significantly impaired when H3K4me3 peptide was used as substrate (33). This suggests that H3K4me3 is less likely an upstream mark of H3K9 methylation. However, MLL1 SET domain has been shown to be capable of methylating H3K4 in the presence of H3K9 methylation (34). Therefore, H3K4me3 can be enzymatically created in H3K9me3-rich regions to establish a bivalent H3 “K4me3-K9me3” methylation pattern for robust Spindlin1 recruitment as well as the maintenance of a “poised” chromatin state.

The above molecular setup is also consistent with the occurrence of H3K9me3/2 erasers that harbor H3K4me3 reader activity, such as JMJD2A and PHF8. Conceivably, to release target genes from a poised state, additional read-and-erase mechanism may be required to resolve the bivalent H3 “K4me3-K9me3” pattern and to recruit other downstream H3K4me3 effectors so as to fully activate gene expression. In the interim, as a maternal factor and a nucleolar protein, Spindlin1 might function as a pioneering reader that could spike in H3K9me3/2-enriched heterochromatic regions in response to H3K4me3 so as to balance gene expression and silencing in processes of early embryogenesis and metabolic stress response. Such a hypothesis awaits in-depth functional dissections in future studies.

## Experimental procedures

### Protein and peptide preparation

Human Spindlin1 (45–262 or 50–262) was cloned on a pGEX-6P vector with an N-terminal GST tag. Recombinant proteins were overexpressed in *Escherichia coli* BL21 (DE3) strain. After induction with 0.2 mm isopropyl β-d-thiogalactoside (IPTG) at 16°C in Luria-Bertani (LB) medium overnight, the cells were harvested and suspended in buffer (200 mm NaCl, 20 mm Tris, pH 8.0). After cell lysis and centrifugation, the supernatant was loaded onto a GST affinity column and washed with lysis buffer. Then the bound proteins were subjected to on-column cleavage by PreScission Protease overnight. The tag-free Spindlin1 was collected for further separation by an anion-exchange QHP column followed by a Superdex 75 gel-filtration column (GE Healthcare). Purified peak fractions were concentrated to 25 mg/ml in 150 mm NaCl, 20 mm Tris, pH 8.0, aliquoted, and stored at −80°C for future use.

All Spindlin1 mutants were generated by the QuikChange Site-Directed Mutagenesis strategy. All mutant Spindlin1 proteins were purified using the same procedure as described above.

All synthetic histone H3 peptides (>95% purity) were purchased from Scilight Biotechnology LLC.

### Crystallization

Spindlin1_45–262_ and H3_1–20_ “K4me3-K9me3/2” were mixed in molar ratio of 1:2 and incubated at 4°C for 1 h. The crystallization trial of the Spindlin1-H3 “K4me3-K9me3/2” complex was performed using the sitting-drop vapor diffusion method. Drops were generated by mixing 200 nl of complex solution with 200 nl of reservoir solution at 18°C. Crystals were obtained under the following buffer conditions: 3.6 m sodium formate, 3% DMSO for Spindlin1-H3 “K4me3-K9me2” complex and 2.8 m sodium acetate, pH7.0, for Spindlin1-H3 “K4me3-K9me3” complex, respectively. Crystals were picked up and soaked in the reservoir solution containing 30% (v/v) glycerol cryo-protectant and flash-frozen in liquid nitrogen for further data collection.

### Data collection and structure determination

Diffraction data were collected at beamline BL17U at Shanghai Synchrotron Radiation Facility. All diffraction datasets were indexed, integrated, and merged using the HKL2000 suite (RRID:SCR_015023). Crystal structures were solved by molecular replacement in CCP4 suite using the published Spindlin1 structure (PDB ID: 2NS2) as a search model. Model building and refinement were performed with COOT (35) and PHENIX (36), respectively. Data processing and structural refinement statistics are summarized in Table 1.

### Isothermal titration calorimetry

Calorimetric experiments were conducted at 25°C with a MicroCal PEAQ-ITC instrument (Malvern Instruments). The Spindlin1_50–262_ samples were dialyzed against the buffer containing 20 mm Hepes Na, pH 7.5, 150 mm NaCl. Protein concentration was determined by UV absorption at 280 nm. Peptides were quantified by weighing on a scale. About 200 µl sample was titrated with 17 successive injections of the corresponding peptide in syringe. Acquired titration curves were fitted and analyzed with the program Origin 7.0 using the One Set of Binding Sites model.

### Thermal shift assay

The TSA was performed with a CFX96 real-time PCR instrument (Bio-Rad). 40 μm Spindlin1_50–262_ was mixed with 60 μm of the corresponding histone peptide and 5× Sypro Orange (Invitrogen). All solutions were prepared in 25 µl of the same buffer (20 mm Hepes Na, pH 7.5, 150 mm NaCl) and incubated at 4°C for 1 h. During TSA assays, samples were heated from 25°C to 90°C at a rate of 0.5°C per minute. Protein denaturation progress was monitored by increased fluorescence signal of Sypro Orange, which captures the exposure of the protein hydrophobic core during thermal unfolding. Changes in the fluorescence signal were recorded and analyzed by the software CFX-Manager (Bio-Rad). When 50% of the protein is denatured, the folding and unfolding states of the protein are in dynamic equilibrium, and the corresponding temperature is the protein's melting temperature.

### ChIP, ChIP-Seq, and quantitative PCR (qPCR)

HEK293 cells were transiently transfected with FLAG-Spindlin1 using Liposome 3000. The ChIP assay was performed as follows. Briefly, cells were crosslinked with 1% formaldehyde for 10 min at 25°C, and the reaction was stopped with 125 mm glycine. After chromatin sonification and centrifugation, the supernatant was aliquoted and subjected to immunoprecipitation overnight at 4°C using ChIP-grade FLAG beads, 4 μg of H3K4me3 and H3K9me3 antibody, respectively. Immune complexes were incubated for 4 h with 50 μl of a mix of Protein A/G agarose at 4°C. Then samples were washed and treated at 65°C overnight for reverse crosslinking. Lastly, the resultant DNA was further eluted and purified for qPCR. The primer sequences used for qPCR analyses are listed here: rDNA loci promoter Forward: 5′-GACCAGTTGTTCCTTTGAGG-3′; rDNA loci promoter Reverse: 5′-GACAGCTTCAGGCACCGCGA-3′; BMP2 (chr20:6767412-6768399) loci Forward: 5′-CAGAGTCCC-CGCGAGGGTCC-3′; BMP2 (chr20:6767412-6768399) loci Reverse: 5′-CGCGCGGCCGCCAAGGGGGG-3′; MIER2 (chr19:344068-345065) loci Forward: 5′-ATAAAGGGTCC-CGGGCTTGT-3′; MIER2 (chr19:344068-345065) loci Re-verse: 5′-TCTCAGAAGAGTTGGACCAG-3′.

For ChIP-Seq, the sequencing libraries were constructed with VAHTS Universal DNA Library Prep Kit (ND607) and sequenced on Illumina HiSeq-PE150 system. We first used software FastQC to check the quality of data, then used bowtie2 to map the data to hg38 genome. After converting the data format to bam (binary alignment/map), we did the peak calling using MACS2, with the q-value cutoff of 0.05 for FLAG-Spindlin1 and 0.1 for H3K4me3 and H3K9me3. The downstream analysis of called peaks was done using R package ChIP seeker.

## Data availability

The atomic coordinates and structure factors of Spindlin1-H3 “K4me3-K9me3” and Spindlin1-H3 “K4me3-K9me2” (codes 7BQZ, and 7BU9) have been deposited in Protein Data Bank. The ChIP-Seq data sets were deposited in GEO database (accession No. GSE147785).

## References

[1] Jenuwein T., Allis C.D. (2001). Translating the histone code. Science.

[2] Musselman C.A., Lalonde M.E., Côte J., Kutateladze T.G. (2012). Perceiving the epigenetic landscape through histone readers. Nat. Struct. Mol. Biol.

[3] Taverna S.D., Li H., Ruthenburg A.J., Allis C.D., Patel D.J. (2007). How chromatin-binding modules interpret histone modifications: Lessons from professional pocket pickers. Nat. Struct. Mol. Biol.

[4] Li Y., Wen H., Xi Y., Tanaka K., Wang H., Peng D., Ren Y., Jin Q., Dent S.Y., Li W., Li H., Shi X. (2014). AF9 YEATS domain links histone acetylation to DOT1L-mediated H3K79 methylation. Cell.

[5] Zhao D., Zhang X.J., Guan H.P., Xiong X.Z., Shi X.M., Deng H.T., Li H.T. (2016). The BAH domain of BAHD1 is a histone H3K27me3 reader. Protein Cell.

[6] Mi W.Y., Zhang Y., Lyu J., Wang X.L., Tong Q., Peng D.N., Xue Y.M., Tencer A.H., Wen H., Li W., Kutateladze T.G., Shi X.B. (2018). The ZZ-type zinc finger of ZZZ3 modulates the ATAC complex-mediated histone acetylation and gene activation. Nat. Commun.

[7] Jiang L., Smith J.N., Anderson S.L., Ma P., Mizzen C.A., Kelleher N.L. (2007). Global assessment of combinatorial post-translational modification of core histones in yeast using contemporary mass spectrometry. LYS4 trimethylation correlates with degree of acetylation on the same H3 tail. J. Biol. Chem.

[8] Fischle W., Tseng B.S., Dormann H.L., Ueberheide B.M., Garcia B.A., Shabanowitz J., Hunt D.F., Funabiki H., Allis C.D. (2005). Regulation of HP1-chromatin binding by histone H3 methylation and phosphorylation. Nature.

[9] Bernstein B.E., Mikkelsen T.S., Xie X., Kamal M., Huebert D.J., Cuff J., Fry B., Meissner A., Wernig M., Plath K., Jaenisch R., Wagschal A., Feil R., Schreiber S.L., Lander E.S. (2006). A bivalent chromatin structure marks key developmental genes in embryonic stem cells. Cell.

[10] Rugg-Gunn P.J., Cox B.J., Ralston A., Rossant J. (2010). Distinct histone modifications in stem cell lines and tissue lineages from the early mouse embryo. Proc. Natl. Acad. Sci. U. S. A.

[11] Voigt P., Tee W.W., Reinberg D. (2013). A double take on bivalent promoters. Genes Dev.

[12] Matsumura Y., Nakaki R., Inagaki T., Yoshida A., Kano Y., Kimura H., Tanaka T., Tsutsumi S., Nakao M., Doi T., Fukami K., Osborne T.F., Kodama T., Aburatani H., Sakai J. (2015). H3K4/H3K9me3 bivalent chromatin domains targeted by lineage-specific DNA methylation pauses adipocyte differentiation. Mol. Cell.

[13] Yuan X., Feng W., Imhof A., Grummt I., Zhou Y. (2007). Activation of RNA polymerase I transcription by Cockayne syndrome group B protein and histone methyltransferase G9a. Mol. Cell.

[14] Feng W., Yonezawa M., Ye J., Jenuwein T., Grummt I. (2010). PHF8 activates transcription of rRNA genes through H3K4me3 binding and H3K9me1/2 demethylation. Nat. Struct. Mol. Biol.

[15] Huang Y., Fang J., Bedford M.T., Zhang Y., Xu R.M. (2006). Recognition of histone H3 lysine-4 methylation by the double Tudor domain of JMJD2A. Science.

[16] Simon M.D., Chu F., Racki L.R., de la Cruz C.C., Burlingame A.L., Panning B., Narlikar G.J., Shokat K.M. (2007). The site-specific installation of methyl-lysine analogs into recombinant histones. Cell.

[17] Zhu Q., Ramakrishnan M., Park J., Belden W.J. (2019). Histone H3 lysine 4 methyltransferase is required for facultative heterochromatin at specific loci. BMC Genomics.

[18] Oh B., Hwang S.Y., Solter D., Knowles B.B. (1997). Spindlin, a major maternal transcript expressed in the mouse during the transition from oocyte to embryo. Development.

[19] Wang J.X., Zeng Q., Chen L., Du J.C., Yan X.L., Yuan H.F., Zhai C., Zhou J.N., Jia Y.L., Yue W., Pei X.T. (2012). SPINDLIN1 promotes cancer cell proliferation through activation of WNT/TCF-4 signaling. Mol. Cancer Res.

[20] Wang W., Chen Z., Mao Z., Zhang H., Ding X., Chen S., Zhang X., Xu R., Zhu B. (2011). Nucleolar protein Spindlin1 recognizes H3K4 methylation and stimulates the expression of rRNA genes. EMBO Rep.

[21] Choi J.W., Zhao M.H., Liang S., Guo J., Lin Z.L., Li Y.H., Jo Y.J., Kim N.H., Cui X.S. (2017). Spindlin 1 is essential for metaphase II stage maintenance and chromosomal stability in porcine oocytes. Mol. Hum. Reprod.

[22] Zhang P., Cong B., Yuan H., Chen L., Lv Y., Bai C., Nan X., Shi S., Yue W., Pei X. (2008). Overexpression of spindlin1 induces metaphase arrest and chromosomal instability. J. Cell. Physiol.

[23] Sun M., Li Z., Gui J.F. (2010). Dynamic distribution of Spindlin in nucleoli, nucleoplasm and spindle from primary oocytes to mature eggs and its critical function for oocyte-to-embryo transition in gibel carp. J. Exp. Zool. A Ecol. Genet. Physiol.

[24] Franz H., Greschik H., Willmann D., Ozretić L., Jilg C.A., Wardelmann E., Jung M., Buettner R., Schüle R. (2015). The histone code reader SPIN1 controls RET signaling in liposarcoma. Oncotarget.

[25] Su X., Zhu G., Ding X., Lee S.Y., Dou Y., Zhu B., Wu W., Li H. (2014). Molecular basis underlying histone H3 lysine-arginine methylation pattern readout by Spin/Ssty repeats of Spindlin1. Genes Dev.

[26] Yang N., Wang W., Wang Y., Wang M., Zhao Q., Rao Z., Zhu B., Xu R.M. (2012). Distinct mode of methylated lysine-4 of histone H3 recognition by tandem Tudor-like domains of Spindlin1. Proc. Natl. Acad. Sci. U. S. A.

[27] Zhao Q., Qin L., Jiang F., Wu B., Yue W., Xu F., Rong Z., Yuan H., Xie X., Gao Y., Bai C., Bartlam M., Pei X., Rao Z. (2007). Structure of human Spindlin1. Tandem tudor-like domains for cell cycle regulation. J. Biol. Chem.

[28] Wang C., Zhan L., Wu M., Ma R., Yao J., Xiong Y., Pan Y., Guan S., Zhang X., Zang J. (2018). Spindlin-1 recognizes methylations of K20 and R23 of histone H4 tail. FEBS Lett.

[29] Zhang X., Zhu G., Su X., Li H., Wu W. (2018). Nucleolar localization signal and histone methylation reader function is required for SPIN1 to promote rRNA gene expression. Biochem. Biophys. Res. Commun.

[30] Ruthenburg A.J., Li H., Patel D.J., Allis C.D. (2007). Multivalent engagement of chromatin modifications by linked binding modules. Nat. Rev. Mol. Cell Biol.

[31] Clackson T., Wells J.A. (1995). A hot spot of binding energy in a hormone-receptor interface. Science.

[32] Wang C., Liu X., Gao Y., Yang L., Li C., Liu W., Chen C., Kou X., Zhao Y., Chen J., Wang Y., Le R., Wang H., Duan T., Zhang Y. (2018). Reprogramming of H3K9me3-dependent heterochromatin during mammalian embryo development. Nat. Cell Biol.

[33] Binda O., LeRoy G., Bua D.J., Garcia B.A., Gozani O., Richard S. (2010). Trimethylation of histone H3 lysine 4 impairs methylation of histone H3 lysine 9: regulation of lysine methyltransferases by physical interaction with their substrates. Epigenetics.

[34] Patel A., Vought V.E., Swatkoski S., Viggiano S., Howard B., Dharmarajan V., Monteith K.E., Kupakuwana G., Namitz K.E., Shinsky S.A., Cotter R.J., Cosgrove M.S. (2014). Automethylation activities within the mixed lineage leukemia-1 (MLL1) core complex reveal evidence supporting a “two-active site” model for multiple histone H3 lysine 4 methylation. J. Biol. Chem.

[35] Emsley P., Cowtan K. (2004). Coot: Model-building tools for molecular graphics. Acta Crystallogr. D Biol. Crystallogr.

[36] Adams P.D., Afonine P.V., Bunkóczi G., Chen V.B., Davis I.W., Echols N., Headd J.J., Hung L.W., Kapral G.J., Grosse-Kunstleve R.W., McCoy A.J., Moriarty N.W., Oeffner R., Read R.J., Richardson D.C. (2010). PHENIX: A comprehensive Python-based system for macromolecular structure solution. Acta Crystallogr. D Biol. Crystallogr.

